# Supplementary material for: Non-invasive decision support for NSCLC treatment using PET/CT radiomics
Source: Nat Commun. 2020 Oct 16;11:5228. doi: 10.1038/s41467-020-19116-x (PMC7567795; doi:10.1038/s41467-020-19116-x)
Supplement: Supplementary file 1 — Supplementary Information [file 41467_2020_19116_MOESM1_ESM.pdf]

SUPPLEMENTARY INFORMATION:

Non-invasive decision support for NSCLC treatment using PET/CT Radiomics

Mu et al.

Supplementary Methods

Inclusion criteria for training, test, TKI-treated and ICI-treated cohorts

For the development of EGFR mutation status prediction model, patients with informed consent were accrued from the Shanghai Pulmonary Hospital (SPH), Shanghai, China and he fourth Hospital of Hebei Medical University (HBMU), Hebei, China, between January 2017 and June 2018 with following inclusion criteria were included: 1) histologically confirmed primary NSCLC; 2) pathological examination of EGFR expression; 3) PET/CT scans were obtained; 4) baseline clinical characteristics (including age, sex, stage, histology, and smoking history) were available. The exclusion criteria included: 1) No EGFR status in record; 2) Pre-treatment was received before Bx; 3) Interval between PET/CT imaging and biopsy (Bx) for immunohistochemistry (IHC) exceeded one month. Based on these inclusion and exclusion criteria, 616 patients were identified and subsequently assigned to a training cohort (training, N =429) and validation cohort (test, N = 187) according to the ratio of 70/30.

For the external test of EGFR prediction and the prognostic value of EGFR-TKI treatment, all the 102 patients with histologically NSCLC who were evolved in a prospective 18F-MPG study (ClinicalTrials.gov: NCT02717221) were enrolled in this study. After applying the exclusion criteria including 1) Claustrophobia, pregnancy, lactation and metal implants in the thorax; 2) No 18FDG-PET/CT before TKI, 72 patients were identified as the external test cohort.

For the distinct cohorts to predict patient outcomes of immunotherapy, all the 447 patients with histologically confirmed advanced stage (stage IIIB and IV) NSCLC who were treated with immunotherapy (anti-PD-L1 or anti-PD-1) between June 2011 and June 2019 at HLM were enrolled. After applying the exclusion criteria including 1) No PET/CT images during the interval (less than 3 months) of the last treatment (or diagnosis) and the start of immunotherapy; 2) other treatment were performed during the interval; 3) follow-up time was less than 6 months; 4) received anti-PD(L)1 antibodies combined with chemotherapy, 149 patients were finally identified.

In total, PET/CT Images and clinical data of 837 NSCLC patients curated from four institutions were analyzed to train and test a deep learning model.

Details of the training of the deep learning model

Preparation of the input images

Pipeline of input images generation is shown in Supplementary Fig. S9. In clinical practice, a non-contrast CT scan was acquired first and then PET scan was acquired subsequent. The PET and CT images were co-registered on the same machine by scanner software. Thus, almost all cases of this study are already registered. A few cases had minor misalignment due to respiratory motion. For these cases, an experienced nuclear medicine radiologist manually adjusted the alignment using ITK-SNAP. Then a square or an irregular box that was close to the boundary of the tumor was delineated by the experienced nuclear medicine radiologist. After resampling with bicubic spline interpolation, dilation of the smallest square mask including the selected region, and resize using cubic spline interpolation, the PET region of interest (ROI) and CT ROI were obtained keeping the entire tumor and its peripheral region with the same size (64×64). Subsequently, the fusion images were calculated through the α-fusion equation:

$$I_{FUSE} = I_{PETnorm} + I_{CTnorm}, \qquad (1)$$

where $I_{PETnorm}$ and I_{CTnorm} are the normalized PET and CT pixel-wise image data by z-score normalization, which means the ROI image is subtracted by the mean intensity value and divided by the standard deviation of the ROI image intensity. The fusion ROI was further standardized by z-score normalization, and constructed a 3-channel hyper-image together with the normalized PET ROI and normalized CT ROI. This hyper-image was used as the input of the SResCNN model. Z-score normalization is performed before inputting to the deep learning model to reduce the effect of different equipment and different reconstruction parameters. Because of the big difference of the central slice and peripheral slices, only the slices with the area larger than the 30% of the maximum area of this patient were regarded as valid input images and were used as the input of the deep learning model. The area here means the area of the smallest square including the selected region (Supplementary Fig. 9c). Finally, 13,583 training ROI-based hyper-images were generated for training.

Structure of the SResCNN network

The 2D SResCNN is based on several residual blocks with 3-channel input images, which is similar to the well-known Resnet18 network with fewer filters for each layer. Given single resolution may not be optimal and depends on the scale of the objects within the image, multi-resolution CNN model was proposed and proved to have significantly better performance[1, 2]. Therefore, the concept of multi-resolution was further incorporated into the architecture,

which was shown in Supplementary Fig. S8. Specifically, the architecture was comprised with three convblocks (including a 3 × 3 convolutional layer followed by a batch normalization layer and a rectified linear unit (ReLU) activation layer) for three different resolutions of the input hyper-images, 8 residual blocks (Resblock), and one fully connected layer. Finally, a softmax activation layer was connected to the last fully connected layer, which was used to yield the prediction probabilities of nodule candidates. Additionally, one dropout layer with probability of 0.5 was added to the fully connected layers. The determination of this architecture was provided in the following Determination of architectures of CNN network section.

Training of the SResCNN network

The training of the model focuses on the optimization of the parameters of the SResCNN model to build a relationship between PET/CT images and EGFR mutation status (positive: 1 or negative: 0). Binary cross entropy was employed as the loss function, while the Adam optimizer was used with an initial learning rate = 0.0001, beta_1=0.9, beta_2=0.999. The learning rate was reduced by a factor of 5 if no improvement of the loss of the validation dataset was seen for a 'patience' number (n=10) of epochs.

The number of the filters, the number of resolutions, the learning rate and the batch size was determined according the predictive performance on the validation cohort using grid search method.

In order to reduce the risk of overfitting, several techniques were deployed. 1) Augmentation: During the training, augmentation including width/height-shift, horizontal/vertical-flip, rotation and zoom were used to expand the training dataset to improve the ability of the model to generalize. 2) Regularization: L2 regularization was used, which added a cost to the loss function of the network for large weights. As a result, a simpler model that was forced to learn only the relevant patterns in the training data would be obtained. 3) Dropout: Dropout layer, which would randomly set output features of a layer to zero during the training process, was added. 4) Early stop: During training, the model is evaluated on the validation dataset after each epoch. The training was stopped after waiting an additional 30 epochs since the validation loss started to degrade.

Application of the SResCNN network

The generated hyper-image was input into the SResCNN model after z-score normalization, and a deeply learned score (DLS) representing the EGFR mutation positivity could be yielded

after a sequential activation of convolution and pooling layers. To develop a robust prediction, all valid slices of each patient were fed into the SResCNN model and the average DLSs with equal weight for each slice was regarded as the final EGFR positive probability of the tumor.

Determination of architectures of CNN network

To select the optimal architecture, we first compared the ResNet18 with a similar architecture but smaller number of filters for each layer (referred as 1-resolution model later, shown in Supplementary Fig. 10 (a)). Trained with our tumor images, the ResNet18 achieved AUCs of 0.88 (95%CI: 0.85, 0.91), 0.78 (95%CI: 0.72, 0.85) and 0.70 (95%CI: 0.57, 0.83) in the training, validation and external test cohorts, respectively. Compared to the 1-resolution model (AUC: training: 0.84, 95%CI: 0.80, 0.87; validation: 0.78, 95%CI: 0.72, 0.85; test: 0.71, 95%CI: 0.59, 0.84), the AUC is higher in the training cohort, but nearly the same in the validation cohorts and slightly lower in the test cohort (Supplementary Fig. 11). This suggests that greater number of filters would not increase the predictive performance, but does increase the risk of overfitting for the task in our study. Additionally, during the training of Resnet18, it took 200 seconds for training each epoch, which was ten times longer than training 1-resolution model. Therefore, the ResNet18-similar architecture with fewer filters for each layer is more appropriate in our study.

Further, in order to validate the necessary of multi-resolution and determine the number of resolutions, architectures with different number of resolutions were trained (Supplementary Fig. 10) and tested on the conditions that the structures of other convolutional layers were kept the same. From the performance shown in Supplementary Fig. 11, when the number of resolution was less than 4, the predictive performance was improved in the training and validation cohorts with the increase number of different resolution. When the number of resolution was 4, though predictive performance was improved in the training cohort, no improvement was found for validation cohort. In order to keep the architecture with fewer parameters, we used 3 different resolutions in this work. Additionally, the advantage of multi-resolution architecture was also proved in the external test cohort, which has more advanced stage cases wither larger tumor volume. That is to say, the multi-resolution architecture is more independent on the scale of the objects within the image.

Based on the above comparison, the current SResCNN network was arrived at and used in this work.

Radiomic quality score (RQS)

Radiomics is a rapidly maturing field in machine learning. To rigorously assess the quality of study design, Lambin et al. developed a 36-point “Radiomics Quality Score” (RQS) metric that evaluates 16 different key components[3]. The full list of criteria is described in Supplementary Table S12, which shows that the current study had an RQS of 16. To put this in perspective, a recent meta-analysis[4] analyzed 77 radiomics publications and documented that the mean ±S.D. RQS across all studies was 9.4±5.6, indicating that the current study is in the upper 20 percentage of radiomics study designs.

Supplementary Figures

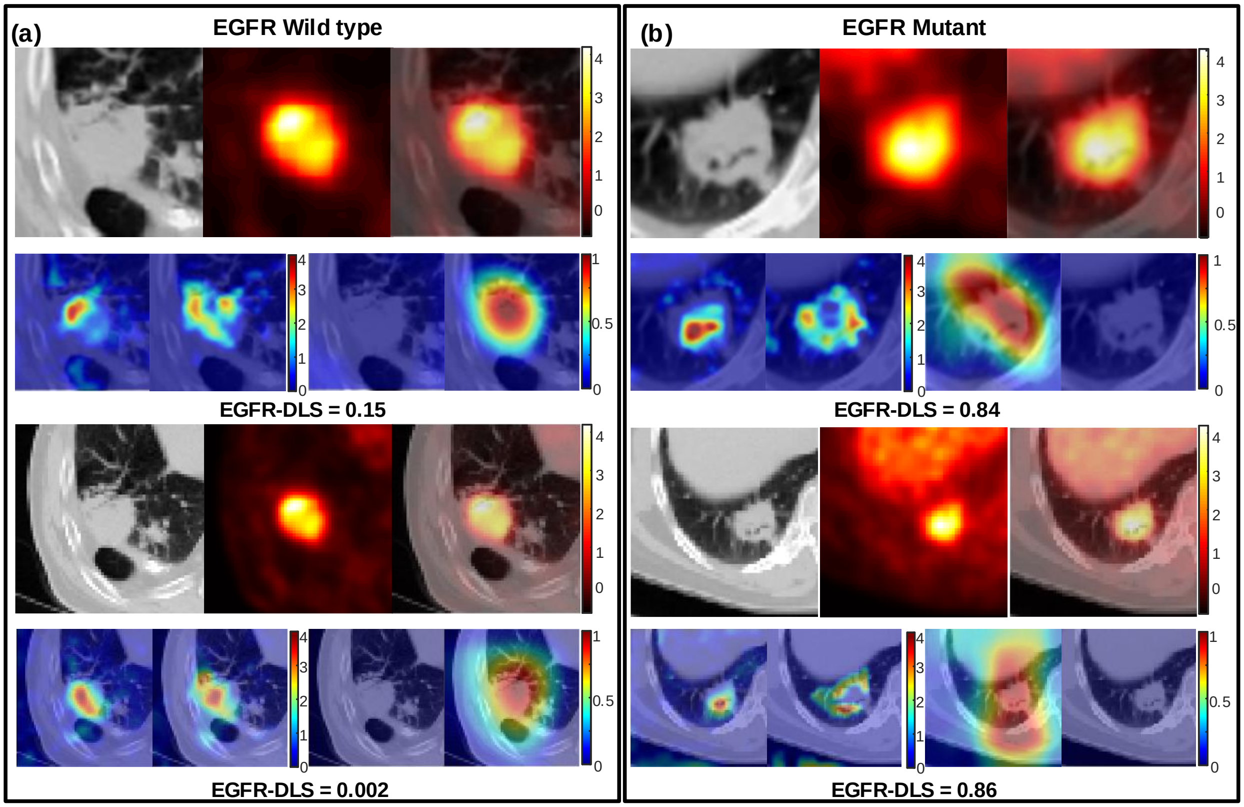

Supplementary Fig. 1. Visualization of the model using different ROIs of the same patients in Fig. 3. (a) and (b) are the patients with wild-type EGFR and EGFR L858 mutant, respectively, which are corresponding to the patients (c) and (d) in Fig. 3. The first lines are the original input ROIs, and the second line show the two of the activation maps of the fourth ResBlocks, the positive filter and the negative filter generated with the original input ROI. The third and fourth lines are the input ROIs with more organs/tissues included, and the corresponding activation maps and positive/negative filter.

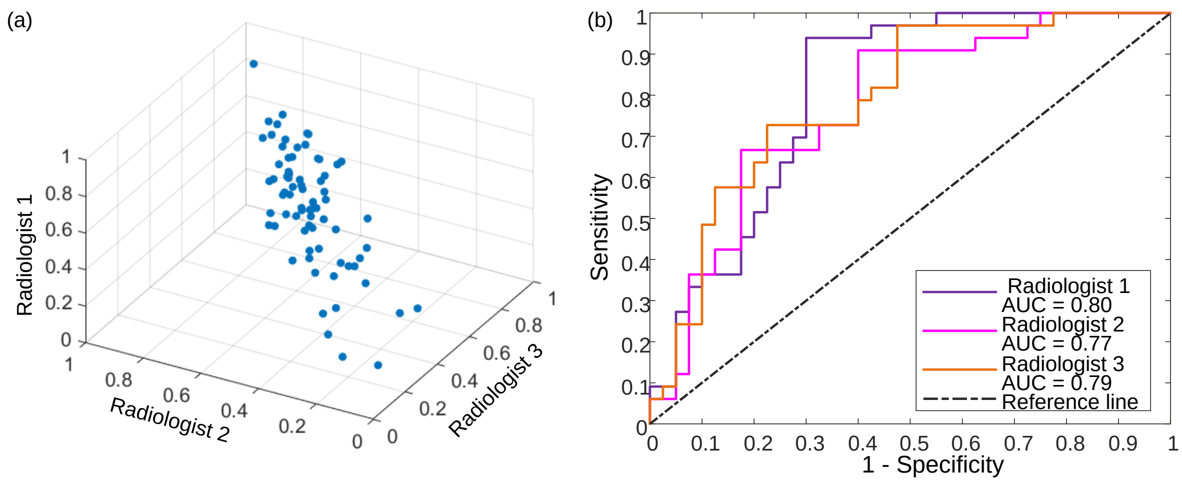

Supplementary Fig. 2. Distribution of EGFR-DLS among different radiologists' delineation. (a) is the correlation of EGFR-DLS among different radiologist's delineation; (b) is the ROC curves of the EGFR-DLSs obtained with different radiologist's delineation.

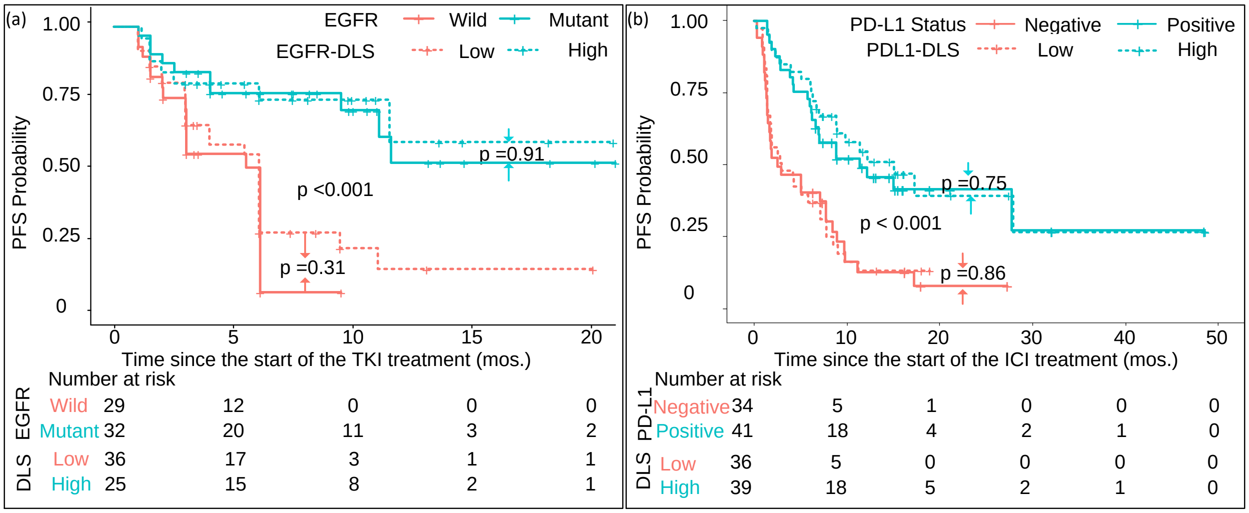

Supplementary Fig. 3. Comparison of progression survival between the molecular biomarkers (EGFR and PD-L1) and image-based biomarkers (EGFR-DLS and PDL1-DLS). (a) is the progression survival of TKI-treated patients with *EGFR* mutation status relative to the EGFR mutation and EGFR-DLS (cutoff:0.5). (b) is the progression survival of ICI-treated patients with PD-L1 status relative to the PDL1-DLS (cutoff:0.54) and PD-L1 status. Note. *p* value was from log rank test.

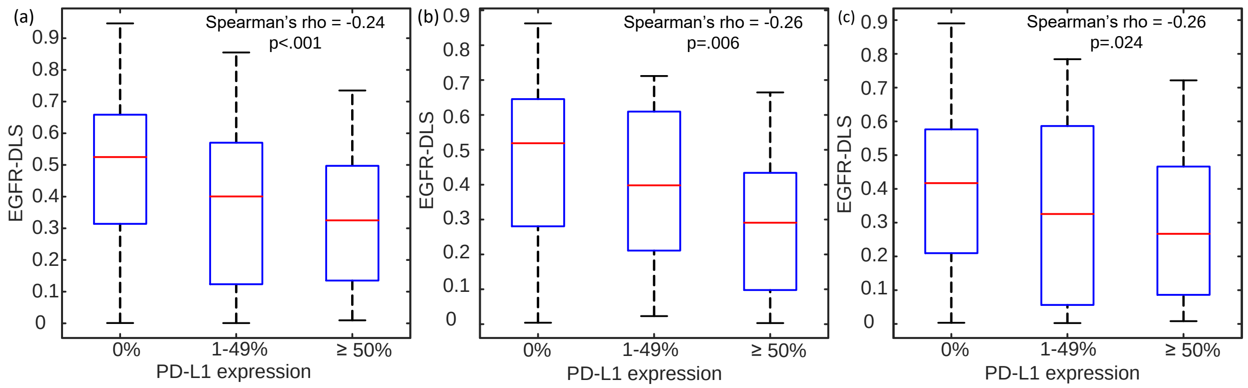

Supplementary Fig. 4. Correlation between EGFR-DLS and PD-L1 expression. (a), (b) and (c) are the EGFR-DLS distribution across different PD-L1 expression in the training, test and HLM ICI-treated sub-cohorts, respectively. In the box plots, the central line represents the median, the bounds of box the first and third quartiles, and the whiskers are the interquartile range. In (a), n = 191, 42, and 34 for 0%, 1-49% and ≥50% groups, respectively. In (b), n = 80, 15, and 17 for 0%, 1-49% and ≥50% groups, respectively. In (c), n = 34, 19, and 22 for 0%, 1-49% and ≥50% groups, respectively.

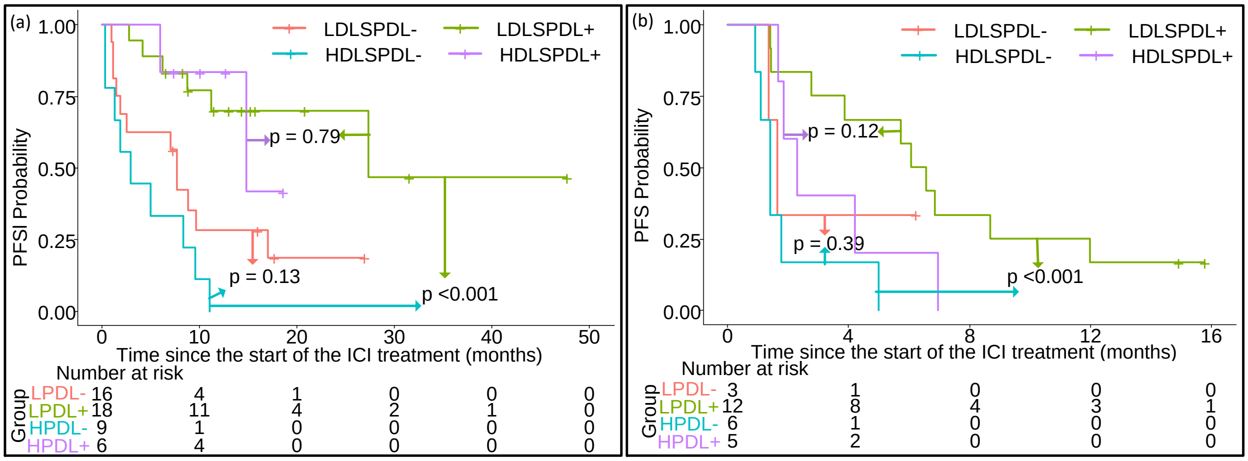

Supplementary Fig. 5. Prognostic value of the EGFR-DLS in the ICI-treated cohorts of different histology type. (a) is the progression survival of adenocarcinoma patients relative to the EGFR-DLS (cutoff:0.5) and PD-L1 status. (b) is the progression survival of squamous cell carcinoma patients relative to the EGFR-DLS (cutoff:0.5) and PD-L1 status. Comparisons of the above progression survival curves were performed with a two-sided log-rank test.

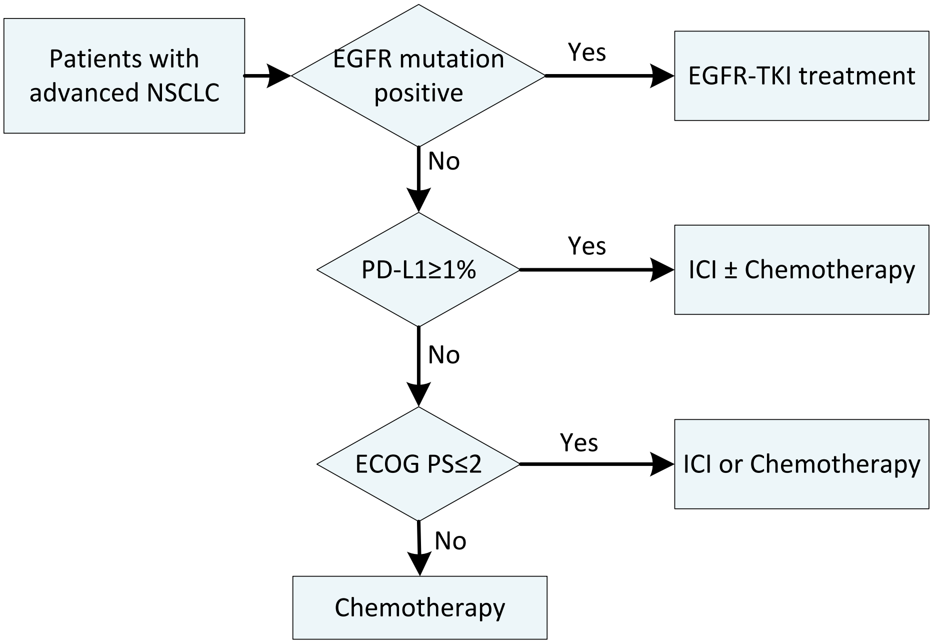

Supplementary Fig. 6. NCCN Guideline Version 2.2020 for Non-Small Cell Lung Cancer.

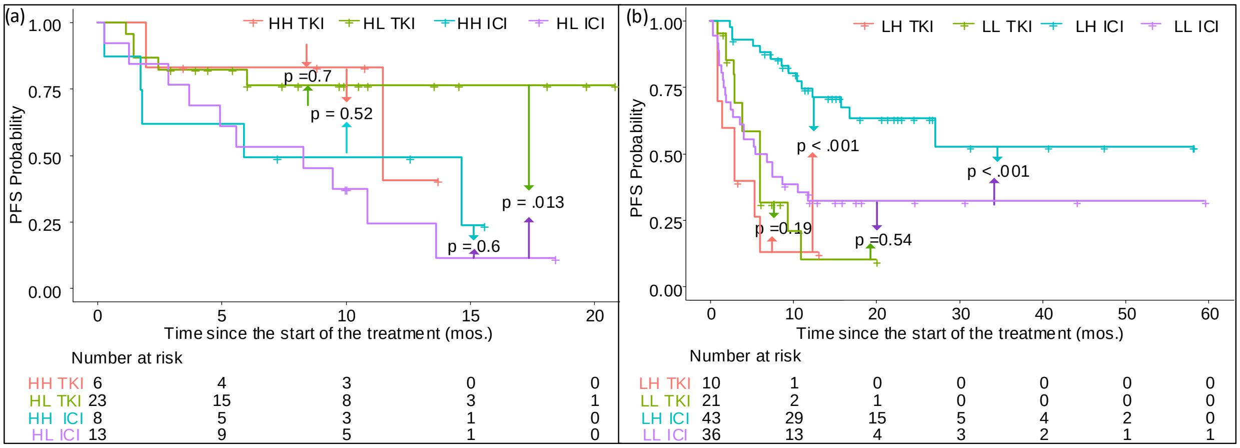

Supplementary Fig. 7. Predictive value of the EGFR-DLS and PDL1-DLS in the combined TKI-treated and ICI-treated cohorts with adenocarcinoma. (a) Progression free survival of patients with high EGFR-DLS. (b) Progression free survival of patients with low EGFR-DLS. H and L refer to higher and lower than median scores, with first letter referring to EGFR-DLS and second to PDL1-DLS. TKI and ICI refer to patients treated with EGFR inhibitors or immune checkpoint inhibitors, respectively. Comparisons of the above progression survival curves were performed with a two-sided log-rank test.

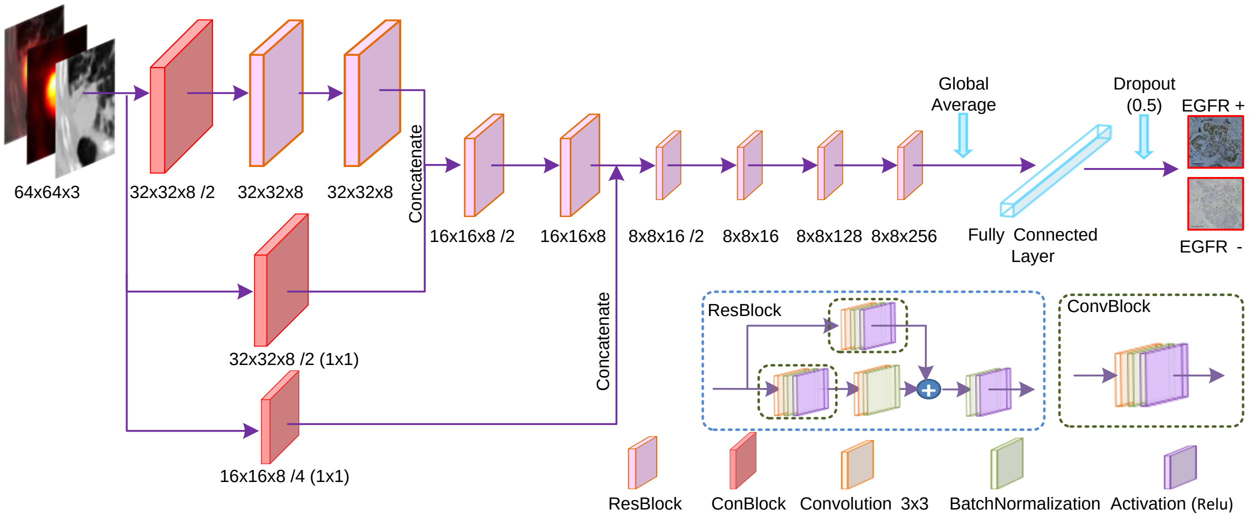

Supplementary Fig. 8. Illustration of the SResCNN model. This model is composed of convolutional layers, batch normalization, pooling, and drop out layers. Note. /2 and /4 mean the convolution layer of the Convblock with stride of 2 and 4, respectively. 1x1 and 3x3 mean the kernel size, without speculation, the default kernel size is 3x3.

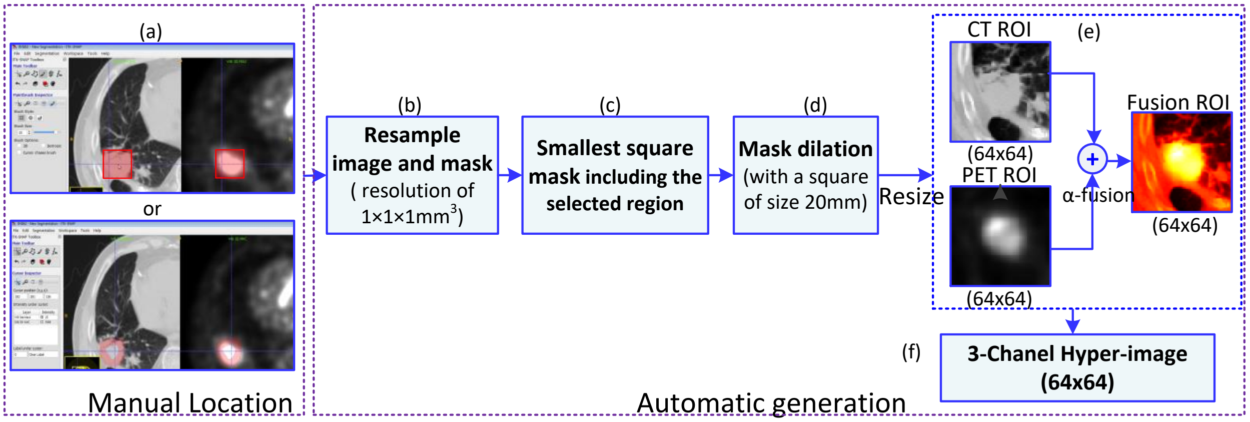

Supplementary Fig. 9. Illustration of the generation of the input hyper-image. A square or an irregular box, which was close to the boundary of the tumor, was delineated manually in ITK software firstly, and then the hyper-image was generated after resampling (cubic interpolation), dilation and fusion automatically.

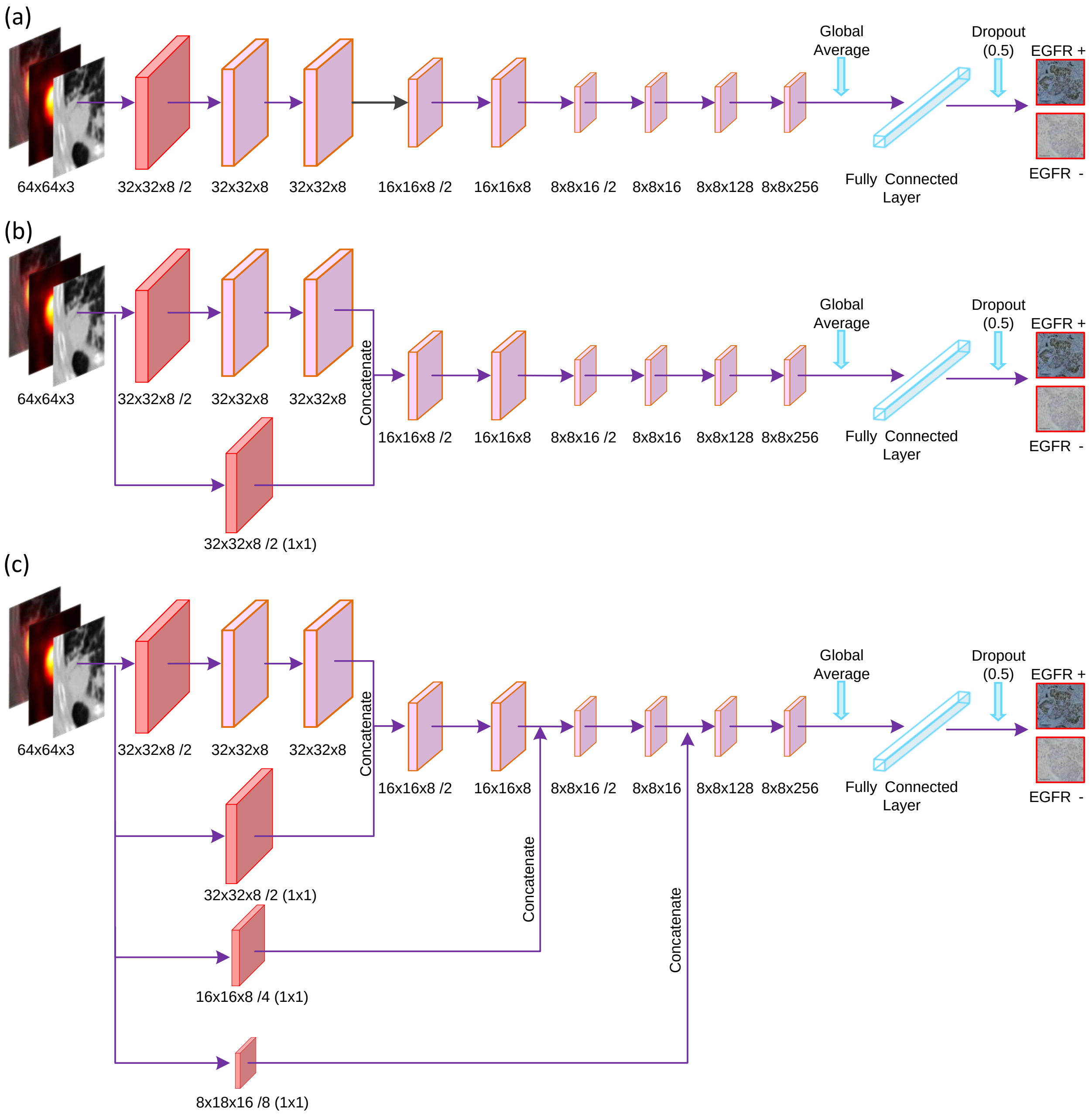

Supplementary Fig. 10. Illustration of the different CNN model. (a) is 1-resolution model with 1 resolution with image size of 64x64; (b) is 2-resolution model with 2 resolutions with image size of 64x64, and 32x32; (c) is 4-resolution model with 4 resolutions with image size of 64x64, 32x32, 16x16 and 8x8.

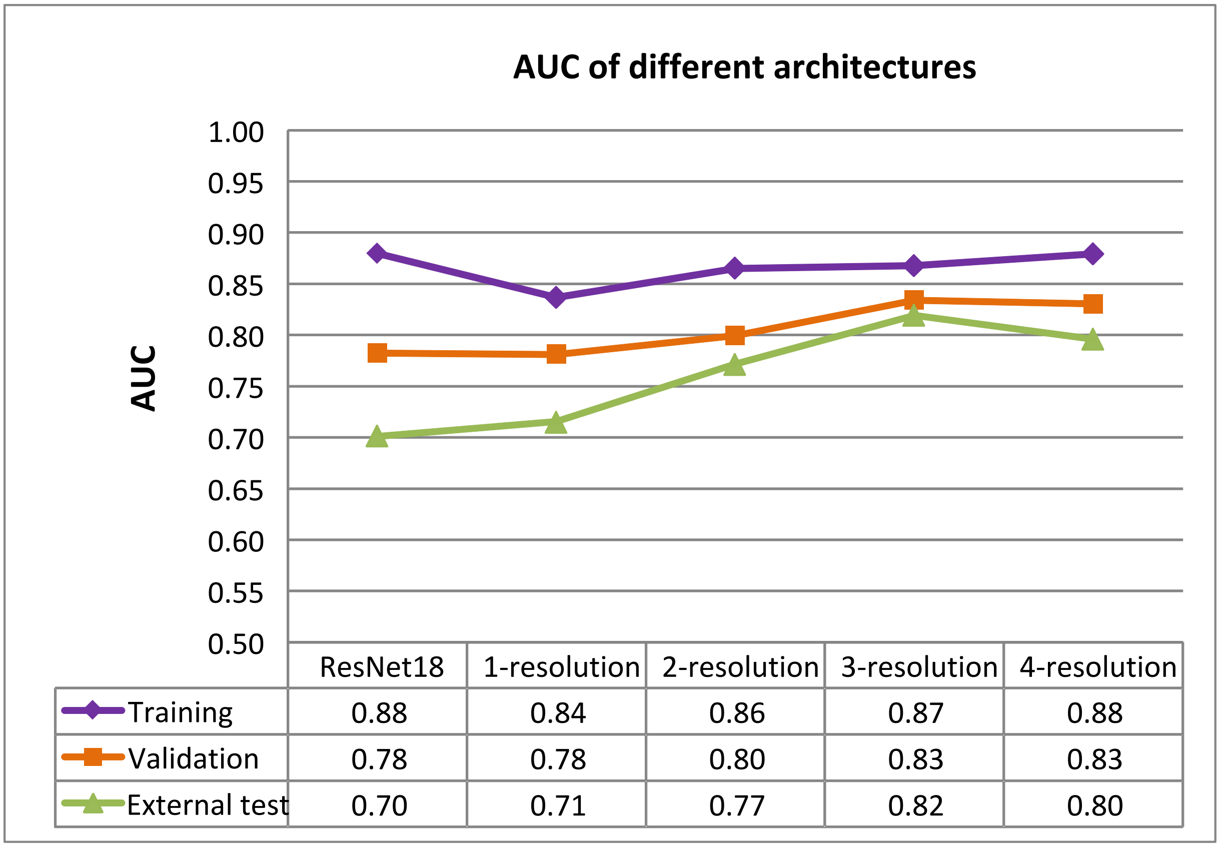

	ResNet18	1-resolution	2-resolution	3-resolution	4-resolution
Training	0.88	0.84	0.86	0.87	0.88
Validation	0.78	0.78	0.80	0.83	0.83
External test	0.70	0.71	0.77	0.82	0.80

Supplementary Fig. 11. Comparison of different architectures based on predictive performance. 1-resolution means the model with only 1 resolution with image size of 64x64; 2-resolution means the model with 2 resolutions with image size of 64x64, and 32x32; 3-resolution means the proposed model in this work with 3 different resolutions with image size of 64x64, 32x32 and 16x16; and 4-resolution means the model with 4 resolutions with image size of 64x64, 32x32, 16x16 and 8x8. Detailed architectures were provided in Supplementary Fig. 10. Each point represents the performance of the corresponding model in the corresponding cohort.

Supplementary Tables

Supplementary Table 1. EGFR predictive performance of different models

	Training cohort	Validation cohort	HMU test cohort
**AUC**			
SUVmax	0.62 (0.56, 0.67)	0.69 (0.61, 0.77)	0.50 (0.35, 0.65)
CS	0.78 (0.74, 0.82)	0.78 (0.72, 0.84)	0.70 (0.58, 0.82)
EGFR-DLS	0.86 (0.83, 0.90)	0.83 (0.78, 0.89)	0.81 (0.72, 0.92)
CMS	0.88 (0.85, 0.91)	0.88 (0.84, 0.93)	0.84 (0.74, 0.93)
**Accuracy**			
SUVmax	58.04 (53.85,62.24)	72.19 (65.78,78.07)	53.85 (43.08,64.62)
CS	72.49 (68.07,76.69)	72.73 (66.31,79.14)	64.62 (53.85,76.92)
EGFR-DLS	81.12 (77.39, 84.62)	81.82 (76.22,86.63)	78.46 (68.50, 87.69)
CMS	82.28 (78.79 85.78)	82.89 (77.01,88.24)	80.00 (70.77,89.23)
**Sensitivity**			
SUVmax	34.33 (27.36,41.29)	52.00 (40, 62.67)	72.22 (58.33,86.11)
CS	78.11 (71.64,83.58)	76.00 (65.33,85.33)	72.22 (58.33,86.11)
EGFR-DLS	84.58 (79.86,89.05)	90.67 (84, 97.33)	69.44 (55.56,83.33)
CMS	83.58 (78.61,88.56)	90.67 (84, 97.33)	69.44 (55.56,83.33)
**Specificity**			
SUVmax	78.95 (73.68,84.21)	85.71 (78.57,91.96)	31.03 (13.79, 8.28)
CS	67.54 (61.40, 73.68)	70.54 (62.50,78.57)	55.17 (37.93, 2.41)
EGFR-DLS	78.07 (72.81,83.33)	75.89 (67.86,83.93)	89.66 (77.67, 100)
CMS	81.14 (76.11,85.96)	77.68 (69.64,84.82)	93.10 (82.76, 100)

Note. Cutoffs for CS, EGFR-DLS and CMS are 0.5. Cutoff for SUVmax is 5 (according to ROC curves of training cohort). CS is short for clinical signature; CMS is short for combined EGFR-DLS and clinical signature.

Supplementary Table 1. Logistic regression analysis of factors for EGFR prediction

Training cohort	Univariate		Clinical signature		Combined signature	
	Odds Ratio(95% CI)	p	Odds Ratio (95% CI)	p	Odds Ratio (95% CI)	p
Age	0.99 (0.99-1.00)	0.17				
Sex	1.11(0.98-1.26)	0.096	2.44 (1.30-4.59)	0.006		
Stage	0.96 (0.85-1.01)	0.088				
Histology	0.75(0.64-0.87)	<.001	0.093(0.03-0.27)	0.006	0.16 (0.052-0.52)	0.002
Smoke	0.30(0.22-0.42)	<.001	0.45 (0.23-0.85)	0.014	0.24 (0.14-0.42)	<.001
SUVmax	0.97 (0.95-0.99)	0.001				
EGFR-DLS	19.52(11.9-32.02)	<.001			14.53 (8.50-24.86)	<.001
Constant			4.71	0.063	2.71	0.13

Validation cohort	Univariate		Clinical signature		Combined signature	
	Odds Ratio(95% CI)	p	Odds Ratio(95% CI)	p	Odds Ratio (95% CI)	p
Age	1.00 (0.97-1.03)	0.93				
Sex	7.74(3.99-15.03)	<.001	5.07(2.54-10.13)	<.001	5.91(2.54-13.77)	<.001
Stage	0.92 (0.71-1.21)	0.57				
Histology	0.036(0.01-0.27)	.001	0.094(0.012-0.74)	.025		
Smoke	0.16(0.084-0.31)	<.001				
SUVmax	0.88(0.82-0.94)	<.001	0.93(0.87-1.00)	0.055		
EGFR-DLS	30.58(12.55-74.5)	<.001			25.77(10.06-66.0)	<.001
Constant			1.52	0.93	0.007	<.001

HMU test cohort	Univariate		Clinical signature		Combined signature	
	Odds Ratio(95% CI)	p	Odds Ratio (95% CI)	p	Odds Ratio (95% CI)	p
Age	0.98(0.93-1.03)	0.35				
Sex	2.83(1.03-7.80)	0.044	2.83(1.03-7.80)	0.044		
Stage	1.12 (0.74-1.67)	0.60				
Histology	-	-				
Smoke	0.36(0.13-0.99)	.048				
SUVmax	1.01 (0.94-1.08)	0.81				
EGFR-DLS	19.70(4.91-79.05)	<.001			19.70 (4.91-79.05)	<.001
Constant			0.25	0.095	0.42	0.017

Note., For Sex: male was assigned set as the referent group and for histology, adenocarcinoma was set as the referent group.

Supplementary Table 2. Multivariate Linear regression analysis of EGFR-DLS on the training cohort

	Standardized Coefficients (95% CI)	p	Partial Correlations	Collinearity Tolerance	VIF
Age	0.015 (-0.002~0.003)	0.72	0.017	0.97	1.03
**Sex**	**0.18 (0.024~0.15)**	**0.007**	**0.13**	**0.40**	**2.50**
Stage	-0.037 (-0.027~0.011)	0.41	-0.040	0.89	1.12
**Histology**	**-0.31(-0.26~-0.14)**	**<.001**	**-0.30**	**0.74**	**1.35**
Smoke	-0.044(-0.084~0.042)	0.51	-0.032	0.39	2.55
**SUVmax**	**-0.14(-0.010~0.002)**	**0.005**	**-0.14**	**0.78**	**1.28**

Note., CI, confidence interval; VIF, variance inflation factor. Parameters in bold were significant in multivariate linear regression analysis. All variance inflation factor (VIF) <5 proved no collinearity among parameters. For Sex: male was assigned 1 and female was assigned 2; for histology, adenocarcinoma was assigned 1 and squamous cell carcinoma was assigned 2.

Supplementary Table 3. Response prediction of EGFR-DLS in TKI-treatment

	TKI-patients	
	EGFR-DLS (median, IQR)	AUC (95%CI, *p*)
Objective response identification		
PR/CR	0.53 (0.33-0.64)	0.67 (0.54-0.80, p=0.019)
SD/PD	0.39 (0.14-0.52)	
Controlled disease identification		
PR/CR/SD	0.52 (0.30-0.63)	0.68 (0.55-0.81, p=0.012)
PD	0.38 (0.12-0.48)	

Note. IQR is short for Interquartile range, and CI is short for confidence interval. *p* values were calculated using two-sided non-parametric Mann-Whitney- U-Test.

Supplementary Table 4. Univariate cox analysis of risk factors for PFS on the independent TKI-treated and ICI-treated cohorts

	TKI-patients		ICI-patients	
	Hazard ratio (95% CI)	*p*	Hazard ratio (95% CI)	*p*
Age	1.03(0.99-1.08)	0.099	0.99 (0.98-1.01)	0.79
Sex	0.84 (0.43-1.64)	0.61	0.73 (0.48-1.11)	0.14
Stage	1.07 (0.80-1.42)	0.65	1.02 (0.71-1.46)	0.92
Histology(baseline)	1.29 (0.45-3.67)	0.64	**2.19 (1.45-3.31)**	**<.001**
Smoke	1.17 (0.59-2.30)	0.66	0.95(0.42-2.15)	0.89
SUVmax	0.95 (0.90-1.02)	0.97	1.00 (0.98-1.03)	0.93
**EGFR-DLS**	**0.24 (0.11-0.57)**	**<.001**	**2.33 (1.51-3.60)**	**<.001**
PD-L1 status	NaN		**0.39 (0.22-0.68)**	**0.001**

Note., For Sex: male was assigned set as the referent group and for histology, adenocarcinoma was set as the referent group. *P* values were derived from Cox proportional hazards model.

Supplementary Table 5. Relationship between Response of ICI-treatment and EGFR-DLS

	EGFR-DLS		EGFR-DLS			
			High		Low	
	High (N=39)	Low (N=110)	PD-L1 + (N=11)	PD-L1 - (N=15)	PD-L1 + (N=30)	PD-L1 - (N=19)
Clinical benefit, NO. (%)						
DCB	13 (33.33)	74 (67.27)	6 (54.55)	3 (20.00)	23 (76.67)	11 (57.89)
NDB	26 (66.66)	36 (32.73)	5 (45.45)	12 (80.00)	7 (23.33)	8 (42.11)
Hyper progression, NO. (%)						
Hyperprogression	13 (33.33)	18 (16.36)	2 (18.18)	9 (60.00)	2 (6.67)	7 (36.84)
non-hyperprogression	26 (66.66)	92 (83.64)	9 (81.82)	6 (40.00)	28 (93.33)	12 (63.16)

Supplementary Table 6. ICI-treated patients' outcomes stratified by EGFR-DLS and histology subtypes

Histology	ADC		SCC	
	High EGFR-DLS (N=20)	Low EGFR-DLS (N=79)	High EGFR-DLS (N=19)	Low EGFR-DLS (N=31)
Median (IQR), months	7.85 [3.32,11.85]	10.73 [4.47,18.25]	2.30 [1.47,4.80]	6.57 [2.09,12.96]
HR [95%CI]	1.87 [1.01,3.48]		2.74 [1.45, 5.17]	
p (cox)	0.048*		0.002*	
p (K-M)	0.09		0.001*	

Note., : *p* (cox) values were derived from two-sided Cox proportional hazards model, while *p* (K-M) was determined with a two-sided log-rank test. * means *P* value <.05.

Supplementary Table 7. ICI-treated patient outcomes stratified by EGFR-DLS and PD-L1 expression

PD-L1 expression	PD-L1 positive		PD-L1 negative	
	High EGFR-DLS (N=11)	Low EGFR-DLS (N=30)	High EGFR-DLS (N=15)	Low EGFR-DLS (N=19)
PFS				
Median (IQR), months	6.97 [2.30,14.80]	12.00 [6.07,-]	1.77 [1.10,5.00]	7.67 [1.47,17.00]
HR [95%CI]	1.57[0.64,3.86]		2.28 [1.07, 4.84]	
p (cox)	0.33		0.032*	
p (K-M)	0.32		0.026*	

Note., : *p* (cox) values were derived from two-sided Cox proportional hazards model, while *p* (K-M) was determined with a two-sided log-rank test. * means *P* value <.05.

Supplementary Table 8. ADC ICI-treated patients' outcomes stratified by EGFR-DLS and PD-L1 expression

PD-L1 expression	PD-L1 positive		PD-L1 negative	
	High EGFR-DLS (N=6)	Low EGFR-DLS (N=18)	High EGFR-DLS (N=9)	Low EGFR-DLS (N=16)
PFS				
Median (IQR), months	14.80 [14.80,-]	27.37 [11.2,-]	2.90 [1.30,8.37]	7.67 [1.47,17.00]
HR [95%CI]	1.25 [0.24,6.48]		1.97 [0.81,4.78]	
p (cox)	0.79		0.14	
p (K-M)	0.79		0.13	

Note., : *p* (cox) values were derived from two-sided Cox proportional hazards model, while *p* (K-M) was determined with a two-sided log-rank test. * means *P* value <.05.

Supplementary Table 9. SCC ICI-treated patients' outcomes stratified by EGFR-DLS and PD-L1 expression

PD-L1 expression	PD-L1 positive		PD-L1 negative	
	High EGFR-DLS (N=5)	Low EGFR-DLS (N=12)	High EGFR-DLS (N=6)	Low EGFR-DLS (N=3)
PFS				
Median (IQR), months	2.30 [1.87,4.20]	6.07 [2.77,8.70]	1.40 [1.10,1.77]	1.63 [1.37,-]
HR [95%CI]	2.43[0.75,7.84]		1.96 [0.39, 9.84]	
p (cox)	0.13		0.41	
p (K-M)	0.12		0.39	

Note., : *p* (cox) values were derived from two-sided Cox proportional hazards model, while *p* (K-M) was determined with a two-sided log-rank test. * means *P* value <.05

Supplementary Table 10. Acquisition parameters for the PET/CT imaging for each cohort

Characteristic	Training cohort	Test cohort	HMU cohort	HLM cohort
**Manufacturer, No. (%)**				
SIMENS	220 (51.28)	112 (59.89)	0	28 (18.79)
GE Medical	48 (11.19)	0	72 (100)	99 (66.44)
PHILIPS	161 (37.53)	75 (40.11)	0	22 (14.77)
**Kilovoltage peak(kVp) , No. (%)**				
120	409 (95.34)	187 (100)	72 (100)	125 (91.67)
130	12 (2.80)	0	0	18 (6.25)
140	8 (1.86)	0	0	6 (2.08)
**Current (mA)**				
Median(range)	228 (59-463)	229 (90-407)	138 (98-404)	81.5 (29-299)
**Interval between administration and image acquisition**				
Mean ± SD	90.49 ± 51.76	90.20 ± 48.18	83.48 ±20.38	93.75 ± 23.55
**Dosage Mbq/kg**				
Mean ± SD	4.38 ± 1.0	4.43 ± 1.28	4.91 ±1.40	5.93± 1.69
**PET Slice Thickness**				
Median(range)	5 (3.26 -5)	5 (4-5)	3.27	3.27(3.26-5)
**PET Pixel Spacing**				
Median(range)	4.07(2.73-4.07)	4.07 (4-4.07)	3.65	3.65(2.73-5.47)
**CT Slice Thickness**				
Median(range)	3 (3 -5)	3 (3 -5)	3.75	3.27(3.27-5)
**CT Pixel Spacing**				
Median(range)	0.98(0.98-1.17)	0.98(0.98-1.17)	0.98(0.98-1.37)	1.37(0.98-1.37)

Supplementary Table 11. The criteria and maximal radiomic quality score as well as the actual score of this work

Criteria	Points system	Maximal score	Actual score of this work
Image protocol quality - well-documented image protocols (for example, contrast, slice thickness, energy, etc.) and/or usage of public image protocols allow reproducibility/replicability	+ 1 (if protocols are well-documented)<br>+ 1 (if public protocol is used)	2	1
Multiple segmentations - possible actions are: segmentation by different physicians/algorithms /software, perturbing segmentations by (random) noise, segmentation at different breathing cycles. Analyse feature robustness to segmentation variabilities	1	1	0
Phantom study on all scanners - detect inter-scanner differences and vendor-dependent features. Analyse feature robustness to these sources of variability	1	1	0
Imaging at multiple time points - collect images of individuals at additional time points. Analyse feature robustness to temporal variabilities (for example, organ movement, organ expansion /shrinkage)	1	1	0
Feature reduction or adjustment for multiple testing - decreases the risk of overfitting. Overfitting is inevitable if the number of features exceeds the number of samples. Consider feature robustness when selecting features	−3 (if neither measure is implemented)<br>+ 3 (if either measure is implemented)	3	0
Multivariable analysis with non radiomics features (for example, EGFR mutation) - is expected to provide a more holistic model. Permits correlating /inferencing between radiomics and non radiomics features	1	1	1

Detect and discuss biological correlates - demonstration of phenotypic differences (possibly associated with underlying gene–protein expression patterns) deepens understanding of radiomics and biology	1	1	1
Cut-off analyses - determine risk groups by either the median, a previously published cut-off or report a continuous risk variable. Reduces the risk of reporting overly optimistic results	1	1	1
Discrimination statistics - report discrimination statistics (for example, C-statistic, ROC curve, AUC) and their statistical significance (for example, p-values, confidence intervals). One can also apply resampling method (for example, bootstrapping, cross-validation)	+ 1 (if a discrimination statistic and its statistical significance are reported) + 1 (if a resampling method technique is also applied)	2	2
Calibration statistics - report calibration statistics (for example, Calibration-in-the-large/slope, calibration plots) and their statistical significance (for example, P-values, confidence intervals). One can also apply resampling method (for example, bootstrapping, cross-validation)	+ 1 (if a calibration statistic and its statistical significance are reported) + 1 (if a resampling method technique is also applied)	2	0
Prospective study registered in a trial database - provides the highest level of evidence supporting the clinical validity and usefulness of the radiomics biomarker	+ 7 (for prospective validation of a radiomics signature in an appropriate trial)	7	0

Validation - the validation is performed without retraining and without adaptation of the cut-off value, provides crucial information with regard to credible clinical performance	- 5 (if validation is missing) + 2 (if validation is based on a dataset from the same institute) + 3 (if validation is based on a dataset from another institute) + 4 (if validation is based on two datasets from two distinct institutes) + 4 (if the study validates a previously published signature) + 5 (if validation is based on three or more datasets from distinct institutes)	5	5
Comparison to 'gold standard' - assess the extent to which the model agrees with/is superior to the current 'gold standard' method (for example, TNM-staging for survival prediction). This comparison shows the added value of radiomics	2	2	2
Potential clinical utility - report on the current and potential application of the model in a clinical setting (for example, decision curve analysis)	2	2	0
Cost-effectiveness analysis - report on the cost-effectiveness of the clinical application (for example, QALYs generated)	1	1	0
Open science and data - make code and data publicly available. Open science facilitates knowledge transfer and reproducibility of the study	+ 1 (if scans are open source) +1 (if region of interest segmentations are open source) + 1 (if code is open source) + 1 (if radiomics features are calculated on a set of representative ROIs and the calculated features and representative ROIs are open source)	4	3
Total score		36	16

Supplementary References

[1]. Kawahara, J., & Hamarneh, Multi-resolution-tract CNN with hybrid pretrained and skin-lesion trained layers. In International workshop on machine learning in medical imaging, 164-171 (2016).

[2]. Zuo, W., et al. Multi-resolution CNN and knowledge transfer for candidate classification in lung nodule detection. IEEE Access 7, 32510-32521 (2019).

[3]. Lambin, P., et al. Radiomics: the bridge between medical imaging and personalized medicine. Nature reviews Clinical oncology 14, 749 (2017).

[4]. Park, JE, et al. Quality of science and reporting of radiomics in oncologic studies: room for improvement according to radiomics quality score and TRIPOD statement. Eur Radiol 30, 523-536 (2019).
